# Prognostic factors for regorafenib treatment in patients with refractory metastatic colorectal cancer: A real-life retrospective multi-center study

**DOI:** 10.17305/bb.2023.9253

**Published:** 2023-12-01

**Authors:** Sabin Goktas Aydin, Engin Eren Kavak, Atakan Topcu, Ayberk Bayramgil, Fahri Akgul, Seda Kahraman, Musa Baris Aykan, Yunus Emre Altıntas, Kaan Helvaci, Yuksel Urun, Ahmet Bilici, Mesut Seker, Mehmet Ali Nahit Sendur, Omer Fatih Olmez, Ozgur Acikgoz, Irfan Cicin

**Affiliations:** 1 Department of Medical Oncology, Medical Faculty, Medipol University, Istanbul, Turkey; 2 Department of Medical Oncology, Medical Faculty, Ankara University, Ankara, Turkey; 3 Department of Medical Oncology, Medical Faculty, Bezmialem Vakif University, Istanbul, Turkey; 4 Department of Medical Oncology, Medical Faculty, Trakya University, Edirne, Turkey; 5 Department of Medical Oncology, Medical Faculty, Ankara Yildirim Beyazit University, Ankara, Turkey; 6 Department of Medical Oncology, Gulhane Education and Research Hospital, Ankara, Turkey; 7 Department of Medical Oncology, Medical Faculty, Koc University, Istanbul, Turkey; 8 Department of Medical Oncology, Memorial Ankara Hospital, Ankara, Turkey

**Keywords:** Regorafenib, metastatic colorectal cancer (mCRC), dose escalation, dose interruption, progression-free survival (PFS), overall survival (OS)

## Abstract

Regorafenib, an oral multikinase inhibitor, has improved survival in metastatic colorectal cancer (mCRC) patients who have progressed on standard therapies. Our study aimed to evaluate prognostic factors influencing regorafenib treatment and assess the optimal dosing regimen in a real-life setting. We retrospectively analyzed 263 patients with mCRC from multiple medical oncology clinics in Turkey. Treatment responses and prognostic factors for survival were evaluated using univariate and multivariate analysis. Of the patients, 120 were male and 143 were female; 28.9% of tumors were located in the rectum. *RAS* mutations were present in 3.0% of tumors, while *BRAF*, *K-RAS*, and *N-RAS* mutations were found in 3.0%, 29.7%, and 25.9% of tumor tissues, respectively. Dose escalation was preferred in 105 (39.9%) patients. The median treatment duration was 3.0 months, with an objective response rate (ORR) of 4.9%. Grade ≥ 3 treatment-related toxicity occurred in 133 patients, leading to discontinuation, interruption, and modification rates of 50.6%, 43.7%, and 79.0%, respectively. Median progression-free survival (PFS) and overall survival (OS) were 3.0 and 8.1 months, respectively. *RAS/RAF* mutation (hazard ratio [HR] 1.5, 95% confidence interval [CI] 1.1–2.3; *P* ═ 0.01), pre-treatment carcinoembryonic antigen (CEA) levels (HR 1.6, 95% CI 1.1–2.3; *P* ═ 0.008), and toxicity-related treatment interruption or dose adjustment (HR 1.6, 95% CI 1.1–2.4; *P* ═ 0.01) were identified as independent prognostic factors for PFS. Dose escalation had no significant effect on PFS but was associated with improved OS (*P* < 0.001). Independent prognostic factors for OS were the initial TNM stage (HR 1.3, 95% CI 1.0–1.9; *P* ═ 0.04) and dose interruption/adjustment (HR 0.4, 95% CI 0.2–0.9; *P* ═ 0.03). Our findings demonstrate the efficacy and safety of regorafenib. Treatment line influences the response, with dose escalation being more favorable than adjustment or interruption, thus impacting survival.

## Introduction

Colorectal cancer (CRC) remains one of the largest contributors to cancer mortality and is the third most frequently diagnosed cancer [1]. Advanced-stage CRC is a life-limiting illness, and treatment options are evolving over time. Regorafenib is an option for patients with metastatic CRC (mCRC) who have been previously treated with fluoropyrimidine-, oxaliplatin-, and irinotecan-based chemotherapy and, if appropriate, an anti-vascular endothelial growth factor (VEGF) agent, anti-epidermal growth factor receptor (EGFR) therapy, and molecular-targeted therapy [2]. Regorafenib is an orally active inhibitor of angiogenic (including VEGF receptors [VEGFRs] 1–3), stromal, and oncogenic receptor tyrosine kinases. It is structurally similar to sorafenib and targets a variety of kinases involved in angiogenic and tumor growth-promoting pathways [3].

The CORRECT trial demonstrated the advantage of regorafenib over best supportive care in refractory mCRC [4]. Additionally, the benefit of regorafenib was also demonstrated in the CONCUR trial. Regorafenib was associated with significantly longer median progression-free survival (PFS) (3.2 vs 1.7 months) and overall survival (OS) (8.8 vs 6.3 months) [5]. Moreover, the CONSIGN phase III trial supported the efficacy of regorafenib in this setting [6].

**Figure 1.**
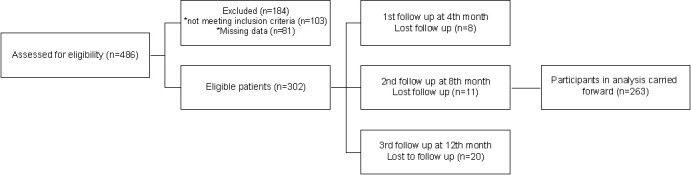
Flowchart of the study.

The initial approved dosage of regorafenib, 160 mg daily for 21 days of a 28-day cycle, may be too high for many patients. However, in the phase II ReDOS trial, a strategy of weekly dose escalation was implemented. This approach began with a daily dose of 80 mg and was gradually increased each week as long as no treatment-related adverse events occurred, with the goal of reaching a daily dose of 160 mg. This alternative dosing strategy allowed more patients to start the third cycle of therapy than those who started directly at 160 mg per day (43% vs 26%). Furthermore, median OS in the dose-escalation cohort showed a positive trend with 9.8 months compared with 6 months in the standard-dose group. Moreover, the incidence of toxicity was more favorable in the dose-escalation cohort [7]. In addition, the tolerability and efficacy of regorafenib have been evaluated in many real-world trials [8–10].

In line with the above information, our aim in the current study was to present multicenter Turkish population data on regorafenib treatment with all aspects, such as toxicity profile, prognostic indicators, and tolerated dosing.

## Materials and methods

Two hundred sixty-three patients diagnosed with mCRC and treated with regorafenib were enrolled in this retrospective and multicenter study between 2018 and 2022. Additional inclusion criteria included the presence of imaging results, a detailed treatment protocol, and toxicity reports. Patients with an ECOG performance status (PS) 3/4 and patients with loss to follow-up were excluded from data analysis. Patients who had synchronous or metachronous tumors were also excluded. The 8th of the tumor, node, metastasis (TNM) staging system of the American Joint Committee on Cancer (AJCC) was used. Patient data were retrospectively obtained from medical records and pathology reports.

A reduced initial dosing strategy was preferred in 105 (39.9%) patients. Thus, the initial dose was 80 mg once daily, which was increased weekly (if tolerated) to the target of 160 mg once daily on days 1–21 of a 28-day treatment cycle. The remaining patients received 160 mg once daily for 21 days of each 28-day cycle. Treatment was continued until disease progression or unacceptable toxicity.

Treatment response and disease progression were evaluated based on clinical and radiological assessment by the treating physician according to the Response Evaluation Criteria for Solid Tumors (RECIST) 1.1 criteria. The objective response rate (ORR) was calculated as the percentage of patients whose tumor regressed (partial response [PR]) or disappeared (complete response [CR]) with regorafenib. Disease control rate (DCR) was the percentage of patients with CR, PR, or stable disease. PFS was defined as the time between regorafenib initiation and disease progression or death. OS was defined as the time between regorafenib initiation and death or last follow-up. Safety was assessed based on the Common Terminology Criteria for Adverse Events (CTCAE) v.5.

### Ethical statement

Written informed consent was obtained from all participants. The local Ethics Committee of Istanbul Medipol University approved the study on 14 April with the decision number E-10840098-772.02-2522. The ethical committee report is available upon request. The Declaration of Helsinki guided the conduct of the study, ensuring ethical standards were upheld in all experimental investigations involving humans or animals.

### Statistical analysis

SPSS 24.0 (SPSS Inc, Chicago, IL, USA) was used for all statistical analyses. Survival analysis, hazard ratios (HR), and 95% confidence intervals (CI) were determined using the Kaplan–Meier method, and survival rates were compared by the log-rank test and stratified according to treatment durations. The influence of prognostic factors was assessed by multivariate Cox proportional analysis. All *P* values were two-sided, and those less than or equal to 0.05 were designated as statistically significant.

## Results

This study enrolled 263 patients with mCRC with a median age of 61 years (range: 24–87 years) (Figure 1). One hundred twenty (54.4%) were male and 143 (45.6%) were female. Seventy-six tumors (28.9%) were localized in the rectum and 186 (71.1%) in the colon. At the time of diagnosis, 2.3%, 9.9%, and 25.1% of patients were diagnosed with stage I, II, and III, respectively. One hundred sixty-five patients were initially diagnosed with stage IV. The median time to metastatic disease was 16.4 months (range: 0–125). Liver, lung, bone, and peritoneal metastases were present in 111 (42.2%), 78 (29.6%), 31 (11.7%), and 64 (24.3%) patients, respectively. One hundred and nine tumors were all-*RAS*-wild-type. In addition, *BRAF*, *K-RAS*, and *N-RAS* mutations were detected in 8 (3.0%), 78 (29.7%), and 68 (25.9%) tumor tissues, respectively (Table 1).

**Table 1:** Patients and tumor characteristics

**Characteristics**	***N* ═ 263**	**(%)**
Age (years), median (range)	61 (24–87)	
*Sex*		
Female	120	54.4
Male	143	45.6
*Tumor localization*		
Right sided	58	22
Left sided	128	49.1
Rectum	76	28.9
*Initial TNM stage*		
I	6	2.3
II	26	9.9
III	66	25.1
IV	165	62.7
Time to metastatic disease, median (range)	16.4 (0–125)	
*Metastatic site*		
Liver	111	42.2
Lung	78	29.6
Peritoneum	64	24.3
Bone	31	11.7
*All-*RAS* wild type*	109	41.4
*BRAF* mutation	8	3.0
*K-RAS* mutation	78	29.7
*N-RAS* mutation	68	25.9
*Treatment line of regorafenib*		
4th line	155	58.9
>4th line	108	41.1
Duration of regorafenib treatment, median (range)	3 (0.5–12.4)	
*CEA before regorafenib treatment, median*		
<19.3 ng/mL	94	37.7
≥19.3 ng/mL	118	44.9

CEA: Carcinoembryonic antigen.

One hundred fifty-five patients received regorafenib in the third- or fourth-line setting. The median duration of treatment was 3 months for all patients (range: 0.5–12.4). The dose escalation strategy was preferred in 105 (39.9%) patients. Thirteen and 63 patients achieved a partial response and stable disease in response to regorafenib, respectively. One hundred forty-seven (55.9%) patients had progression. The ORR was 4.9%. Treatment-related toxicity was observed in 84.7% of patients. The toxicity-related treatment discontinuation, interruption, and modification rates were 50.6%, 43.7%, and 79.0%, respectively. Eleven patients were able to tolerate 160 mg regorafenib daily without interruption or dose adjustment. At the time of progression, the final regorafenib dose was 120 mg daily in 65.7% of patients. Treatment-related grade 3 or higher toxicity occurred in 133 (50.6%) patients. The most common ≥grade 3 toxicities were skin reactions on hands and feet (6.8%), fatigue (4.6%), loss of appetite (4.2%), and hypertension (3.8%). Severe hematologic toxicity and nausea/vomiting were reported in five and six patients, respectively (Table 2).

**Table 2:** Regorafenib response rates with toxicity profile

	***N* ═ 263**	**(%)**
RECIST 1.1		
Partial response	13	4.9
Stable disease	63	24.0
Progressive disease	147	55.9
Objective response rate	4.9%	
Any grade toxicity	223	84.7
Grade 3/4 toxicity	69	26.2
Dose modification	208	79.0
Treatment interruption	115	43.7
Treatment discontinuation	133	50.6
Adverse event (Grade 3/4)		
Fatigue	12	4.6
Loss of appetite	11	4.2
Hand and foot skin reaction	18	6.8
Hypertension	10	3.8
Nausea/vomiting	6	2.9
Diarrhea	2	0.7
Oral mucositis	2	0.7
Increased AST/ALT	3	1.1
Hematological toxicity	5	1.9

RECIST 1.1: Response Evaluation Criteria for Solid Tumors; AST: Aspartate transaminase; ALT: Alanine transaminase.

Patients were categorized according to the presence or absence of dosage escalation. There were no significant differences in age, sex, tumor localization, and metastatic sites between the two groups. However, 61.5% of patients in the dose-escalation group had an *RAS/RAF* mutated tumor, while 47.4% in the standard therapy group had *RAS/RAF* mutations (*P* ═ 0.05). Dose escalation was preferred in 68.6% and 56.9% of patients aged over and under 61 years, respectively (*P* ═ 0.06).

**Figure 2.**
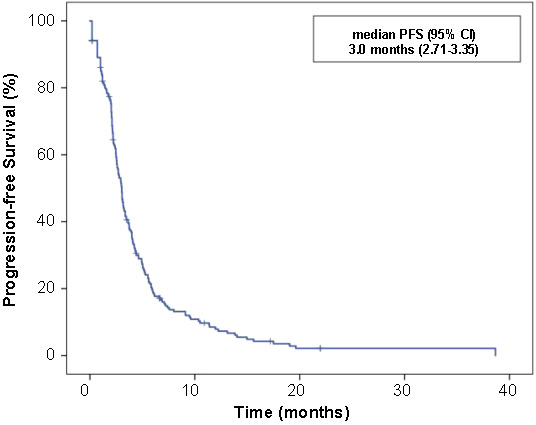
Progression-free survival (PFS) curve with regorafenib treatment in the intention to treat population.

**Figure 3.**
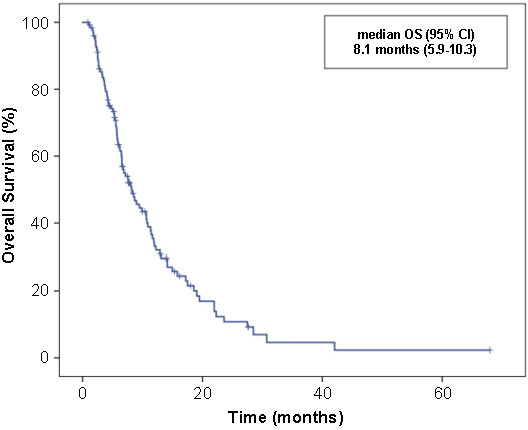
Overall survival (OS) curve with regorafenib treatment in the intention to treat population population.

With a median follow-up of 27.9 months (range: 2.0–98.1), median PFS and OS were 3.0 months (95% CI 2.71–3.35) and 8.1 months (95% CI 5.9–10.3), respectively, in patients treated with regorafenib (Figures 2 and 3).

**Figure 4.**
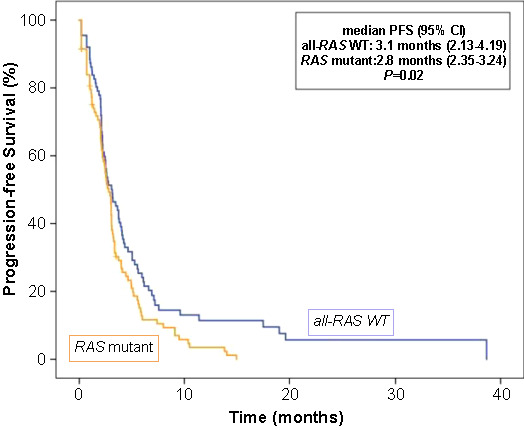
Progression-free survival (PFS) curve according to the *RAS/RAF* mutation status.

In univariate analysis, there was no significant relationship between PFS and age, sex, initial TNM stage, tumor localization, metastatic site, or regorafenib treatment line. Median PFS was significantly better in all-*RAS*-wild-type tumors compared with mutated tumors (3.1 months vs 2.8 months; *P* ═ 0.02) (Figure 4). The median carcinoembryonic antigen (CEA) level before starting treatment with regorafenib was 19.3 ng/mL. The number of patients with CEA < 19.3 ng/mL was 94 (37.7%). The median PFS was significantly worse in patients with ≥ 19.3 ng/mL than in those with CEA < 19.3 ng/mL (2.2 vs 3.6 months; *P* ═ 0.01). Despite the difference was not statistically significant, patients who required treatment interruption or dose adjustment tended to have worse survival than those who did not need any dose adjustment or interruption (2.2 vs 3.3 months; *P* ═ 0.05). Dose escalation had no significant effect on PFS (*P* ═ 0.2).

Multivariate analysis for PFS showed that *RAS/RAF* mutation (HR 1.5, 95% CI 1.1–2.3; *P* ═ 0.01), pre-treatment CEA levels (HR 1.6, 95% CI 1.1–2.3; *P* ═ 0.008) and toxicity-related treatment interruption or dose adjustment (HR 1.6, 95% CI 1.1–2.4; *P* ═ 0.01) were independent significant prognostic factors.

**Figure 5.**
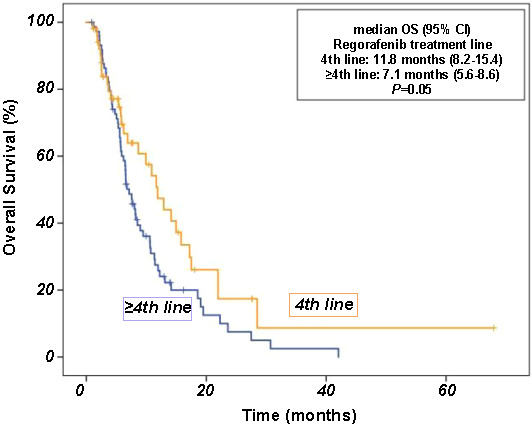
Overall survival (OS) curve according to the treatment line of regorafenib.

**Figure 6.**
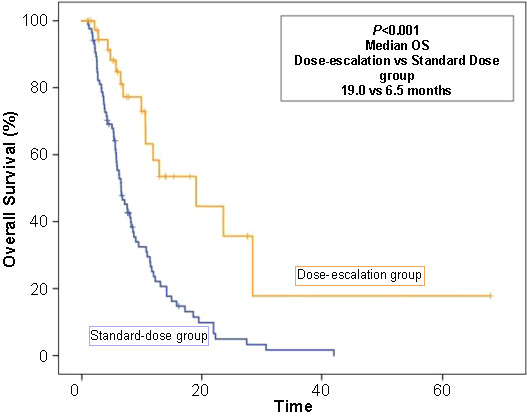
Overall survival (OS) curve according to the initial dosing regimen of regorafenib.

Univariate analysis for OS revealed that initial TNM stage (*P* ═ 0.03), tumor localization (*P* ═ 0.05), regorafenib treatment line (*P* ═ 0.05), and dose interruption (*P* ═ 0.05) were significant prognostic indicators. However, there was no significant relationship between OS and age, sex, median CEA levels, tumor metastatic site, and *RAS/RAF* mutation. Comparing patients over 80 years of age to the younger population, there was no significant difference in survival (*P* ═ 0.06). Compared with later lines of therapy, regorafenib prolonged OS in the fourth line of therapy (median OS 7.1 vs 11.8 months; *P ═* 0.05) (Figure 5). However, significance was not observed when comparing treatment lines 3 vs ≥ 3; statistical significance was observed between 4 and ≥ 4 lines. Controversially, toxicity-related dose adjustment or interruption significantly worsened OS. Additionally, dose escalation was significantly associated with better OS (*P* < 0.001) (Figure 6). Multivariate analysis showed that the independent prognostic factors for OS were initial TNM stage (HR 1.3, 95% CI 1.0–1.9; *P* ═ 0.04) and dose interruption/adjustment (HR 0.4, 95% CI 0.2–0.9; *P* ═ 0.03). The details of prognostic factors for survival are shown in Table 3.

**Table 3:** Multivariate and univariate analysis for progression-free survival and overall survival

	**Progression-free survival**		**Overall survival**	
**Factors**	**Univariate**	**Multivariate**	**Univariate**	**Multivariate**
	***P* value**	***P* value (HR, 95% CI)**	***P* value**	***P* value (HR, 95% CI)**
Age, years (median)	0.8	–	0.9	–
Sex				
Female vs male	0.7	–	0.7	–
Tumor localization	0.9	–	**0.05**	–
TNM stage	0.1	–	**0.03**	**0.04** **(1.3, 1.0–1.9)**
Metastatic site	0.3		0.5	
Liver	0.5		0.8	
Lung	0.1	–	0.6	–
Peritoneum	0.3		0.4	
Bone	0.1		0.2	
*RAS/RAF* mutation	**0.02**	0.01 (1.5, 1.1–2.3)	0.6	
Treatment interruption or dose reduction	**0.05**	0.01 (1.6, 1.1–2.4)	**0.05**	**0.03** **(0.4, 0.2–0.9)**
Dose escalation	0.2	–	**<0.001**	0.70 (0.8, 0.4–1.7)
Pre-regorafenib median CEA	**0.01**	**0.008** **(1.6, 1.1–2.3)**	0.3	**-**

Bold indicates statistical significance. CEA: Carcinoembryonic antigen.

## Discussion

This multicenter study aimed to add data to the current literature and demonstrate the real-world experience with regorafenib and its tolerability and optimal dosing in patients with mCRC. The efficacy and safety of regorafenib have already been published in clinical trials [4–6]. Moreover, several large real-world studies have confirmed the effectiveness of regorafenib in mCRC patients [10–14].

In practice, median OS and treatment duration varied between 5.6–7.0 months and 2.2–2.7 months, respectively, similar to phase III trials [4–6, 11–14]. Small retrospective series showed that median PFS and OS varied between 2.7–3.9 months and 7.6–9.3 months, respectively [14–18]. The first study in Turkish patients by Dane et al. [10], which primarily investigated safety, showed a median PFS of 3.1 months. In our study, the median PFS and OS were 3.0 and 8.1 months, respectively, with a median follow-up of 27.9 months.

The radio-CORRECT study, a retrospective analysis of the CORRECT study, demonstrated that the change in target lesion size after RECIST 1.1 was a predictive factor for regorafenib outcomes [19]. Another subgroup analysis of the CORRECT study revealed that *K-RAS* and *PIK3CA* mutations were favorable prognostic factors for survival [20]. In addition, other subgroup analyses identified several prognostic biomarkers [21]. In real-world experiences, the potential prognostic factors were poor ECOG PS, a short time from the initial diagnosis of metastases to the start of regorafenib treatment (<160 mg), >3 metastatic sites, the presence of liver metastases, and the presence of *K-RAS* mutations [11, 14, 18]. A lung-limited metastatic disease was also associated with better survival rates [22, 23]. CEA is a well-known and recommended prognostic marker in mCRC [24]. Despite this, Unseld et al. [25] found no significant correlation between response rates, PFS and *RAS* status, CEA, and CA19-9 levels. Del Prete et al. [26] analyzed the role of regorafenib treatment line and showed that it was statistically significant for OS. Another study by Aparicio et al. [27] showed that age over 80 years was associated with worse survival. However, our study did not confirm these results in the Turkish population.

Prognostic markers for mCRC patients treated with regorafenib include *RAS/RAF* mutation status, pre-treatment CEA levels, and treatment interruption/dose adjustment. All-*RAS*-wild-type tumors correlated with better PFS, while higher pre-treatment CEA levels (>19.3 ng/mL) and the need for treatment dose adjustment were associated with worse PFS. Initial TNM stage and dose interruption/adjustment were independent prognostic factors for OS in our study.

In contrast to the literature, metastatic site did not affect survival in our cohort [27]. Nevertheless, there is no doubt that these results contribute to the literature by showing prognostic features in the Turkish mCRC population treated with regorafenib.

Despite all these survival benefits, treatment-related toxicities, such as hand-foot skin reactions and fatigue, are major limitations of regorafenib use. Clinical trial data demonstrated that toxicities occur early after regorafenib treatment initiation, usually within the first cycle, and improve rather than worsen over time [28]. Thus, the ReDOS trial compared the dose-escalation strategy (starting dose 80 mg/day orally with weekly escalation in 40 mg increments to 160 mg/day) with the standard-dose strategy (160 mg/day orally for 21 days of a 28-day cycle). The primary endpoint was met, and the most common grade 3–4 adverse events in the treatment groups were fatigue (13% vs 18%), hand-foot skin reaction (15% vs 16%), abdominal pain (17% vs 6%), and hypertension (7% vs 15%). The median OS was 9.8 months in the dose escalation group compared with 6.0 months in the standard-dose group (HR 0.72, 95% CI 0.47–1.10; *P* ═ 0.12) [7] However, dose modification was higher in clinical trials and the REGARD study showed that it was 55% in the Turkish population [10, 29].

The toxicity profile of our cohort was similar to previous studies [4, 5]. However, toxicities might have been underestimated due to the retrospective design. To our knowledge, this is the first study that indicated the impact of dose escalation, dose modifications, and adjustments in a real-world setting. The dose escalation strategy was preferred by 39.9% of our patients. The toxicity-related treatment discontinuation, interruption, and modification rates were 50.6%, 43.7%, and 79.0%, respectively. And only 11 patients could tolerate 160 mg regorafenib daily without interruption or dose adjustment. Median PFS was 2.2 vs 3.3 months in patients who required treatment interruption or dose adjustment compared with those who did not need any dose adjustment or interruption (*P* ═ 0.05). Dose escalation had no significant effect on PFS (*P* ═ 0.2). However, dose escalation and dose interruption/adjustment significantly affected OS (*P* < 0.001 and *P* ═ 0.05, respectively). Moreover, dose interruption/adjustment (HR 0.4, 95% CI 0.2–0.9; *P* ═ 0.03) was an independent prognostic factor for OS in mCRC patients treated with regorafenib.

Our results demonstrate the efficacy and safety of regorafenib in patients with mCRC. Our study contributes to the literature by demonstrating the adverse effects of regorafenib dose modification and interruption on OS. Moreover, it was found that patients who received dose escalation had a higher prevalence of *RAS/RAF*-mutated tumors and a tendency to have a better OS, suggesting the potential benefit of dose escalation in specific subgroups of mCRC patients. Regarding these results, the dose escalation strategy, which has shown significant benefits for OS, could be prioritized in the Turkish population. Our study included 263 patients from multiple oncology clinics, which increases the power of the analysis.

The retrospective and non-randomized design of this study, which was the major limitation of the study, could lead to bias in our results. We did not evaluate the ECOG PS, comorbidities, or characteristics of the patients according to dosing strategies. In addition, ECOG PS data were not available for most patients because of the retrospective nature of our study. A notable finding is the unexpectedly balanced representation of *N-RAS* and *K-RAS* mutations in our cohort. Typically, *N-RAS* mutations are reported to be 10 times less frequent than *K-RAS* mutations. However, in our study, a relatively equal distribution of both types of mutations was observed. This atypical pattern might be influenced by regional genetic variations and the specific ethnic diversity of the Turkish population included in our study. Despite these limitations, we believe that the results of this large study may be helpful in decision making in clinical practice because it analyzes the effects of dose escalation, dose modification, and adjustment in a real-world setting.

## Conclusion

Our survival and toxicity results are consistent with those in the literature. Regorafenib is a safe and effective option for mCRC patients. This study has shown that dose interruption significantly affects survival. Hence, a dose escalation strategy might allow more patients to remain on regorafenib, which significantly correlated with better OS in the Turkish population.
